# Analysis of Dental Prosthetic Treatment in Patients with Cancer Aged 65 Years and Older after Expanded Health Insurance Coverage: A Retrospective Clinical Study

**DOI:** 10.3390/medicina60091509

**Published:** 2024-09-16

**Authors:** Hyo-Jung Kim, Iel-Yong Sung

**Affiliations:** 1 Department of Dentistry, Ulsan University Hospital, University of Ulsan College of Medicine, 25, Daehakbyeongwon-ro, Dong-gu, Ulsan 44033, Republic of Korea; 2 Department of Oral and Maxillofacial Surgery, Ulsan University Hospital, University of Ulsan College of Medicine, 25, Daehakbyeongwon-ro, Dong-gu, Ulsan 44033, Republic of Korea; cmfs65@hotmail.com

**Keywords:** cancer, comorbid illness, dental prosthetic treatment, insurance benefits, unmet dental care needs

## Abstract

*Background and Objectives*: With increases in cancer incidence and the number of cancer survivors, the demand for cancer management is growing. However, studies on dental prosthetic treatment for patients with cancer are rare. We aim to investigate the dental prosthetic treatment in patients with cancer aged ≥65 years after expanded health insurance coverage. *Materials and Methods*: This retrospective study included patients who were treated with implants and removable dentures at Ulsan University Hospital in South Korea between June 2015 and June 2023. Data on age, sex, cancer location, comorbid systemic diseases, number of remaining teeth, dental prosthetic treatment history, type of dental prosthetic treatment, and insurance coverage status were extracted from patient medical records and panoramic radiographs. The influence of multiple variables on dental prosthetic treatment was analyzed using the Chi-square and Fisher’s exact tests. *Results*: The study included 61 patients with cancer (32 men, 29 women; average age: 70.9 years). Among them, 56 (91.8%) had insurance coverage benefits, and 34 (55.7%) received treatments such as implants, removable partial dentures, or complete dentures for the first time. Treatment types included 37 (60.7%) cases of implant prostheses and 24 (39.3%) conventional removable dentures. No statistical differences were observed in the type of dental prosthetic treatment according to sex, age, cancer location, number of systemic diseases, and dental prosthetic treatment history (*p* > 0.05). Patients with <10 remaining teeth received treatment with conventional removable dentures, which was statistically significant (*p* < 0.001). *Conclusions*: Of the 61 patients, 56 (91.8%) received insurance benefits, and 34 (55.7%) underwent dental prosthetic treatment for the first time. Within the limitations of this retrospective study, the expanded health insurance coverage alleviated the unmet demand for dental prosthetic treatment. As cancer prevalence continues to increase, expanding customized health insurance coverage is crucial to meet this demand.

## 1. Introduction

According to the 2022 Korea statistics on causes of death, malignant neoplasms (cancer), heart disease, coronavirus disease 2019, pneumonia, cerebrovascular disease, intentional self-harm (suicide), Alzheimer’s disease, diabetes, hypertension, and liver disease accounted for 67.4% of all deaths, with cancer being the leading cause of mortality in both men and women [1]. Since mortality statistics began to be published in 1983, cancer has been consistently ranked as the leading cause of death in Korea. Over the past 20 years, a substantial increase in cancer survival rates has been noted, with the 5-year survival rates increasing from 42.9% in 1993–1995 to 71.5% in 2016–2020. As the number of cancer survivors continues to rise, there is an increasing demand for comprehensive cancer management strategies [2]. However, oral diseases are usually regarded as optional to treat in older adults owing to the ongoing financial burden associated with the treatment of cancer and other systemic diseases [3].

Tooth loss generally increases with age, influenced by factors such as systemic diseases, medications, psychological effects, and a decline in both the interest in and ability to maintain oral hygiene [4]. A systematic review and meta-analysis reported that the prevalence of tooth loss increased gradually with age and peaked at 65 years of age [5]. Based on the 2020 National Survey of Older Koreans, 36.9% of the older population use dentures, and among them, 39.5% reported discomfort while chewing [6]. Oral health problems are not limited to patients with head and neck cancer [7]. Cancer treatments such as radiation and chemotherapy can cause various oral complications, including mucositis, reduced saliva secretion, dental caries, periodontal diseases, and ultimately, tooth loss [8].

A nationwide survey by the Ministry of Health and Welfare in Korea revealed that 54.9% of individuals aged 65 years and older suffer from two or more chronic conditions [6]. An increase in the number of chronic diseases from none or one to two or more results in a considerable increase in total out-of-pocket medical expenses [9]. Unmet medical needs refer to situations where individuals require medical care and are unable to receive it owing to various reasons [10]. This phenomenon is particularly pronounced among the older [11] and low-income groups [12]. The rate of unmet dental care needs was 3.7%, which is relatively higher than the rate of unmet medical care needs. The reasons for unmet dental care included economic difficulties, minor symptoms, fear of treatment processes, lack of time, mobility issues, and difficulties with hospital appointments or long waiting times [3,6,13].

Oral mastication plays a critical role as the initial step of the digestive system and facilitates the intake of various foods, contributing to the prevention of systemic frailty [14,15]. Therefore, oral health care should be better integrated into medical care owing to its strong correlation with overall health [16]. Nevertheless, the integration of oral health in cancer care for older people to prevent or minimize oral health complications of cancer treatments is uncommon, except in head and neck oncology [3]. Increased dental care utilization has been associated with having dental insurance. Efforts to promote the use of dental care should consider providing cost-effective dental insurance options [17]. Since 2012, the National Health Insurance Service (NHIS) of Korea provided insurance coverage for oral reconstruction procedures, including complete dentures (CD), removable partial dentures (RPD), and implants. This initiative has led to a notable increase in the number of dental visits among older patients with systemic diseases [18]. However, studies analyzing dental treatments in patients with cancer, excluding those with head and neck cancers, remain scarce. In this retrospective study, we aimed to investigate the dental prosthetic treatment in patients with cancer aged 65 years and older after expanded health insurance coverage. The two null hypotheses of this study were as follows: (1) the dental prosthetic treatment history in patients with cancer aged 65 years and older would not be associated with sex, age, cancer location, number of systemic diseases, number of remaining functional teeth, or type of dental prosthetic treatment received, and (2) the type of dental prosthetic treatment in patients with cancer aged 65 years and older would not vary according to sex, age, cancer location, number of systemic diseases, number of remaining functional teeth, or dental prosthetic treatment history.

## 2. Materials and Methods

### 2.1. Study Design

Among patients who were treated with implants and removable dentures at Ulsan University Hospital in South Korea between June 2015 and June 2023, this retrospective clinical study focused on those with cancer aged ≥65 years. This study was approved by the Institutional Review Board of Ulsan University Hospital (IRB protocol no.: 2024-05-009, approved on 17 May 2024). Patient consent was waived owing to the retrospective nature of this study.

### 2.2. Inclusion and Exclusion Criteria

The inclusion criteria were as follows: patients diagnosed with cancer who were aged 65 years and older, those who underwent dental prosthetic treatment in our department, and those who had complete medical data available. The exclusion criteria included patients with cancer who were under 65 years of age, those who did not receive prosthetic treatment in our department, and those with incomplete medical record data. 

### 2.3. Data Collection

Data collection was conducted by a prosthodontist who performed all the prosthetic treatments. A total of 61 patients were included. Sex, age, cancer location, number of systemic diseases, number of remaining functional teeth, dental prosthetic treatment history, type of dental prosthetic treatment received, and insurance coverage status were investigated by reviewing the patients’ medical records and panoramic radiographs.

Based on location, cancers were classified into the following categories: gastrointestinal, genitourinary, head and neck, hematologic, thyroid, breast, and other types of cancers. Patients were divided into groups according to the number of their remaining functional teeth (≥10 or <10). Teeth with severe decay and remaining roots were not considered functional teeth [19]. In addition, patients were categorized based on the number of systemic diseases that they had (<2 or ≥2). Patients were also classified according to the types of dental prosthetic treatment received (implant prostheses or conventional removable dentures).

### 2.4. Data Analysis

The dental prosthetic treatment history was analyzed according to sex, age, cancer location, number of systemic diseases, number of remaining functional teeth, or type of dental prosthetic treatment received. Additionally, the type of dental prosthetic treatment received was investigated based on sex, age, cancer location, number of systemic diseases, number of remaining functional teeth, or dental prosthetic treatment history.

Data were organized using Microsoft Excel 2021 (Microsoft, Redmond, WA, USA). All statistical analyses were performed using IBM SPSS Statistics version 24 (IBM Corp., Armonk, NY, USA). Distributions, percentages, and means are presented as descriptive statistics for all variables. The Chi-square test was used to compare variables. Because of the nonuniform distribution of the data and the small number of patients, the Fisher’s exact test was employed to accurately determine the impact of cancer location on dental prosthetic treatment history and the type of dental prosthetic treatment received. Statistical significance was set at *p* < 0.05.

## 3. Results

The study included 61 patients with cancer (32 men, 29 women) aged 65 to 86 years (average age: 70.9 years). Table 1 presents the baseline characteristics of our cohort. The number of different cancer types are as follows: 19 gastrointestinal, 15 genitourinary, 9 head and neck, 6 hematologic, 5 thyroid, 4 breast, and 3 other types of cancers. Additionally, 30 patients (49.2%) had <2 systemic diseases and 31 (50.8%) had ≥2. Cardiovascular disease was the most common, affecting 39 patients, followed by diabetes mellitus in 19, kidney disease in 8, and stroke in 6.

Of the 61 patients, 24 (39.3%) had <10 remaining functional teeth, and 37 (60.7%) had ≥10. Fifty-six patients (91%) received insurance coverage benefits, with five patients not covered. Five patients received implant overdentures (IODs) that were not covered by insurance.

Of the 61 patients, 34 (55.7%) had no dental prostheses and received treatments such as implants, RPDs, or CDs for the first time (Table 2). No statistical differences were noted in dental prosthetic treatment history according to sex, age, cancer location, number of systemic diseases, number of remaining functional teeth, or type of dental prosthetic treatment (*p* > 0.05).

Among the 61 patients, 37 (60.7%) received implant prostheses, and 24 (39.3%) received removable dentures (Table 3). No statistical differences were noted in the type of dental prosthetic treatment according to sex, age, cancer location, number of systemic diseases, or dental prosthetic treatment history (*p* > 0.05). Significantly, patients with <10 remaining teeth received treatment with removable dentures (*p* < 0.001).

## 4. Discussion

Oral function is essential for nutrient intake and can be restored using dental prosthetic treatments in patients with tooth loss. Dental prostheses reportedly reduced the risk of weight loss among those with tooth loss [20]. Fewer remaining teeth and no dental prosthesis use were linked to social isolation, whereas dental prosthesis use mitigated this especially in those with severe tooth loss [21].

This retrospective study investigated 61 patients with cancer (average age: 70.9 years). In addition to cancer, 54 patients (88.5%) had additional systemic diseases. Of the 61 patients, 56 (91.8%) received insurance coverage benefits, 34 (55.7%) received treatments such as implants, RPD, or CD for the first time, with implant prostheses being more common than conventional removable dentures. No statistical differences were observed in the type of dental prosthetic treatment according to sex, age, cancer location, number of systemic diseases, and dental prosthetic treatment history (*p* > 0.05). However, patients with <10 remaining teeth received treatment with removable dentures (*p* < 0.001). Based on our results, the first null hypothesis was accepted because no statistically significant difference was observed in the dental prosthetic treatment history of patients with cancer based on the variables examined in our study. The second null hypothesis was partially rejected, as a significant difference was found in the type of dental prosthetic treatment received according to the number of functional teeth. Our study suggests that older patients with cancer were less likely to receive dental prosthetic treatments despite having chewing difficulties owing to the loss of multiple teeth and that expanded health insurance coverage led to an increase in dental prosthetic treatments.

With an increase in the number of newly diagnosed cancer cases and patient survival rate, the demand for dental care is also increasing [2]. Among patients with cancer, 29.0% have been reported to have chewing problems and 13.6% encounter speech difficulties, especially among those with lower incomes [22]. Cancer treatments such as chemotherapy, radiotherapy, hematopoietic stem cell transplantation, supportive antiresorptive therapies, and targeted and immune therapies have cytotoxic effects on oral tissues, resulting in various oral complications [3]. Patients with bone metastases from breast or prostate cancer or multiple myeloma often receive high-dose intravenous antiresorptive therapy, increasing their risk of medication-related osteonecrosis of the jaw, thereby contraindicating implant treatments. Decreased saliva secretion, often caused by radiation therapy or polypharmacy, can make wearing CD nearly impossible owing to poor retention and pain caused by the dry denture base rubbing against the sensitive mucosa. Clinical decision making should involve consulting with the patient’s primary care physician, considering the survival rates and quality of life (QoL) [23,24,25]. The use of implants for oral rehabilitation in patients with head and neck cancer is becoming increasingly common [26], improving QoL by enhancing denture retention and reducing tissue load [27]. Despite implants being particularly beneficial for patients with head and neck cancer owing to the difficulty of conventional oral rehabilitation after tumor removal surgery [24], patients did not typically continue with dental reconstruction after mandibular resection, with cost being a major barrier [28]. Meanwhile, in this study, of the nine patients with head and neck cancer, seven received implant prostheses and two received dentures. Except for one patient who received treatment with IODs that were not covered by insurance, all eight patients were covered by insurance and received dental prosthetic treatment for the first time, likely owing to expanded dental coverage.

In this study, among the 61 patients, 23 (37.7%) had one systemic disease other than cancer, and 31 (50.8%) had more than two. Among older individuals with chronic diseases, 1.6% experienced unmet medical needs, primarily attributed to financial burdens (55.9%), physical limitations (31.6%), and time constraints (12.5%) [29]. Oral health care is often neglected compared to overall health [30]. Out-of-pocket expenses can create financial barriers for socioeconomically disadvantaged groups, hindering their access to dental prosthetic treatments and leading to the neglect of oral health [13]. In patients wearing dentures, approximately one-third reported poor fit and retention during meals [31]. A study on oral health-related QoL (OHRQoL) found significant improvements after the implementation of denture health insurance benefits. Before the implementation, 36.2% of individuals had their dentures made at nonmedical facilities owing to cost burdens [32]. However, the expansion of dental insurance has significantly improved accessibility to these treatments [33]. Consistent with the findings of previous studies [30,32], 34 patients (55.7%) had no dental prosthesis despite experiencing chewing discomfort owing to tooth loss. We assumed that owing to the severity differences based on the number of comorbidities in patients with cancer, those with more comorbidities would opt for conventional dentures over invasive implant prostheses. However, no statistical difference was observed in the type of dental prosthetic treatment according to cancer location or number of systemic diseases in this study.

Dental health insurance reform began in mid-2012 with coverage for CDs for those ≥75 years old, later expanding to those ≥65 years old. Since June 2013, RPDs and implants have also been covered under the national health insurance. Initially, the personal copayment for dental services was 50%; however, it was reduced to 30% in 2019. This reduction significantly increased dental service utilization and decreased out-of-pocket expenses for older individuals, improving dental service usage equality and reducing financial burdens [34]. A study revealed that the expansion of insurance coverage for dental prosthetics had a significant impact on older patients with major diseases [35]. A study on insured patients with dentures found that 58.8% of respondents had used dentures for <5 years, and 66.6% had their current dentures for <5 years. This indicates that many had their current dentures newly made after the implementation of medical insurance coverage for dentures in 2012 [36]. Cooray et al. [37] reported that a copayment discount policy increased oral health service utilization among older Japanese. Consistent with the findings of the previous studies, most of the patients in this study received treatment covered by insurance. After receiving the initial treatment that was covered by insurance, participants continued with treatment that was not covered, as the NHIS of Korea currently only covers up to two implants for a lifetime. Seven patients who had previously not used dentures owing to poor adaptation also chose insurance-covered dentures.

Patients with systemic diseases are often excluded from implant treatments to minimize risks, leading many to prefer treatment with RPDs or CDs instead of implants. Seo et al. suggested that, given the higher risk of systemic diseases in older patients and their tendency to avoid invasive interventions, insurance coverage for dentures might be more beneficial than coverage for implants [18]. In this study, 17 out of 24 patients with <10 remaining teeth received treatment with conventional removable dentures. Additionally, 12 out of 24 patients had both or one edentulous arch. However, 37 out of 61 participants (60.7%) were treated with implant prostheses. A study on the utilization rate of implant insurance benefits revealed that the 65–69-year age group exhibited the highest utilization rate, and the utilization rate of dental implants was higher in men than in women [38]. Consistent with previous research [38], the 65–74-year age group, men, and patients with no previous prosthetic history tended to choose implants for their prosthetic treatment, although no statistical significance was found. The high utilization rate of implants is attributed to the inclusion of implant-assisted removable partial dentures (IARPDs) and IODs. A recent study found that men, older adults, less educated individuals, and adults with low socioeconomic status were less likely to have seen a dentist within the past 5 years and more likely to have lost their permanent teeth [39]. Another study reported that unmet health care needs mainly consisted of lack of dental care owing to poor health insurance coverage; furthermore, age was the main factor associated with unmet health care needs [40]. By contrast, in our study, no statistical difference was noted in the type of dental prosthetic treatment according to age and sex owing to insurance benefits.

Dentures are generally undervalued owing to concerns related to convenience, aesthetics, masticatory function, occlusal stability, and oral hygiene maintenance. However, with the advent of various treatment methods using implants, the noninvasive and cost-effective advantages of dentures are enhancing their competitiveness [41]. Distal-extension RPDs in patients with shortened dental arches partially compensate for reduced masticatory performance [42]. IARPDs provide additional support and retention with a few strategically placed implants, limiting the movement of the prostheses. This enhances the comfort, aesthetics, speech, and masticatory function of RPD [43]. Common clinical issues with mandibular CDs, in addition to discomfort, include a lack of retention and stability. IODs are relatively easier to adapt to and offer higher masticatory efficiency than CDs, benefiting from support and retention provided by the implants placed underneath the CD [44]. In a two-period crossover study comparing maxillary implant-retained fixed prostheses with IODs, IODs received significantly higher patient ratings based on patients’ ability to speak and the ease of cleaning [45]. Thomason et al. suggested that using two IODs in the mandible should be the minimum standard for most individuals owing to performance, patient satisfaction, cost, and clinical time [46]. For edentulous mandibles, IODs supported by two or four implants improve OHRQoL outcomes and are considered cost effective [47,48,49]. In this study, of the 14 patients with mandibular edentulism who chose dentures, six were treated with IODs that were not covered by insurance. Currently, the NHIS of Korea covers implants only for patients with partial edentulism. Expanding insurance coverage to include implants for patients with edentulism, which are currently not covered, could significantly improve the OHRQoL for patients with cancer and those in remission.

Much research has been conducted regarding unmet dental care needs in the general population, but studies on the unmet dental care needs of patients with cancer, excluding those with head and neck cancer, are rare. Oral health is closely related to overall health; therefore, attention to the oral health of patients with cancer is necessary.

The retrospective nature of this clinical study introduces certain limitations, including the small number of participants and the lack of an assessment of QoL before and after treatment. The limited sample size and its selection may not fully represent the broader population of patients with cancer aged 65 years and older, which could result in weakness in confirming statistical significance. It is crucial to interpret the findings herein with caution. Future research designs should be standardized, and prospective clinical research with larger sample sizes is warranted to produce reliable and generalizable results.

## 5. Conclusions

Of 61 patients, 56 (91.8%) received insurance benefits, and 34 (55.7%) received removable denture and implant treatment for the first time. Within the limitations of the present retrospective study, the long treatment duration and financial burden of cancer led to unmet dental prosthetic treatment needs, manifesting currently as an increased demand for dental care owing to expanded health insurance coverage. With the rising prevalence of cancer, the demand for dental treatments is also increasing and is expected to grow further in the future. Therefore, expanding customized health insurance coverage is necessary to meet this demand.

## Figures and Tables

**Table 1:** Baseline characteristics of patients.

Characteristics	Patient (*n*)	Ratio (%)
Sex		
Male	32	52.5
Female	29	47.5
Age (y)		
<75	48	78.7
≥75	13	21.3
Cancer site		
Gastrointestinal	19	
Genitourinary	15	
Head and neck cancer	9	
Hematologic	6	
Thyroid	5	
Breast	4	
Others	3	
Comorbid systemic diseases		
None	7	11.5
One systemic disease	23	37.7
Two systemic diseases	22	36.0
Three or more systemic diseases	9	14.8
Number of remaining teeth		
<10	24	39.3
≥10	37	60.7
Dental prostheses		
No	34	55.7
Yes	27	44.3
Dental insurance benefit		
No	5	8.2
Yes	56	91.8

**Table 2:** Dental prosthetic treatment history.

	Prior Dental Prosthetic Treatment			
	Overall	No	Yes	*p*-Value
	(N = 61)	(N = 34)	(N = 27)	
Sex, *n* (%)				0.548 ^†^
Male	32 (52.5)	19 (55.9)	13 (48.1)	
Female	29 (47.5)	15 (44.1)	14 (51.9)	
Age (y), *n* (%)				0.877 ^†^
<75	48 (78.7)	27 (79.4)	21 (77.8)	
≥75	13 (21.3)	7 (20.6)	6 (22.2)	
Cancer site, *n* (%)				0.276 ^‡^
Head and neck	9 (14.8)	7 (20.6)	2 (7.4)	
Others	52 (85.2)	27 (79.4)	25 (92.6)	
No. of comorbid illnesses, *n* (%)				0.883 ^†^
<2	30 (49.2)	17 (50.0)	13 (48.1)	
≥2	31 (50.8)	17 (50.0)	14 (51.9)	
No. of remaining teeth, *n* (%)				0.075 ^†^
<10	24 (39.3)	10 (29.4)	14 (51.9)	
≥10	37 (60.7)	24 (70.6)	13 (48.1)	
Type of treatment, *n* (%)				0.210 ^†^
Dental implant	37 (60.7)	23 (67.6)	14 (51.9)	
Removable denture	24 (39.3)	11 (32.4)	13 (48.1)	

^†^ Chi-square test, ^‡^ Fisher’s exact test.

**Table 3:** Types of prosthetic treatments received.

	Types of Prosthetic Treatments			
	Overall	Dental Implant	Removable Denture	*p*-Value
	(N = 61)	(N = 37)	(N = 24)	
Sex, *n* (%)				0.174 ^†^
Male	32 (52.5)	22 (59.5)	10 (41.7)	
Female	29 (47.5)	15 (40.5)	14 (58.3)	
Age (y), *n* (%)				0.571 ^†^
<75	48 (78.7)	30 (81.1)	18 (75.0)	
≥75	13 (21.3)	7 (18.9)	6 (25.0)	
Cancer site, *n* (%)				0.462 ^‡^
Head and neck	9 (14.8)	7 (18.9)	2 (8.3)	
Others	52 (85.2)	30 (81.1)	22 (91.7)	
No. of comorbid illnesses, *n* (%)				0.918 ^†^
<2	30 (49.2)	18 (48.6)	12 (50.0)	
≥2	31 (50.8)	19 (51.4)	12 (50.0)	
No. of remaining teeth, *n*(%)				<0.001 ^†^
<10	24 (39.3)	7 (18.9)	17 (70.8)	
≥10	37 (60.7)	30 (81.1)	7 (29.2)	
Dental prostheses, *n* (%)				0.210 ^†^
No	34 (55.7)	23 (62.2)	11 (45.8)	
Yes	27 (44.3)	14 (37.8)	13 (54.2)	

^†^ Chi-square test, ^‡^ Fisher’s exact test.

## Data Availability

The data are included in the study, further inquiries can be directed to the corresponding author.
